# Effects of Autotoxicity and Allelopathy on Seedling Growth in Cashew (*Anacardium occidentale* L.)

**DOI:** 10.3390/plants15040583

**Published:** 2026-02-12

**Authors:** Esther Dansoa Tetteh, Kwame Sarpong Appiah, Christiana Amoatey, Clepton Antwi Korsah, Ransford Ampofo, Ernest Kobina Aidan, Yoshiharu Fujii

**Affiliations:** 1 Department of Crop Science, College of Basic and Applied Science, University of Ghana, Legon, Accra P.O. Box LG 44, Ghana; edtetteh97@gmail.com (E.D.T.); camoatey@ug.edu.gh (C.A.); korsahclepton@gmail.com (C.A.K.); ransfordampofo311@gmail.com (R.A.); ernestaidan5@gmail.com (E.K.A.); 2 Faculty of Agriculture, Tokyo University of Agriculture and Technology, Saiwai-Cho 3-5-8, Fuchu 183-8509, Japan

**Keywords:** *Anacardium occidentale*, autotoxicity, seedling growth, continuous cropping obstacle, aqueous extract

## Abstract

Cashew (*Anacardium occidentale* L.), a vital tropical cash crop, may face yield declines in old plantations due to unexplored risks of autotoxicity. This study investigated the allelopathic and autotoxic potential of cashew plant under laboratory and greenhouse conditions. The laboratory bioassays with leaf and stem bark (10–200 mg) demonstrated a strong allelopathic effect, reducing lettuce radicle elongation to 7–46.0% and 9–79% of the control, respectively. Aqueous leaf extract (50 mg/mL) completely inhibited (0%) lettuce seed germination and reduced pepper germination to 42%. However, the root exudate of cashew seedlings did not have any inhibitory effect on the test plants. Greenhouse experiments simulating field litter fall revealed significant autotoxicity in cashew. Cashew seedlings grown in growth media amended with 10% cashew leaf powder exhibited severe growth suppression after 13 weeks, including a reduction in plant height by 58.2% compared to controls. Chlorophyll content, stem girth, and leaf number were also significantly reduced. This study concludes that cashew possesses significant allelopathic properties and a clear potential for autotoxicity, as directly evidenced by the suppressed growth of its own seedlings following the incorporation of leaf powder. These findings identify autotoxicity, mediated through leaf litter decomposition, as a critical risk factor for the replanting success and long-term sustainability of cashew orchards, necessitating further investigation into management strategies.

## 1. Introduction

Allelopathy is a widespread biological phenomenon where plants produce and release bioactive substances through leaching, volatilization, litter fall decomposition, and root exudate into the environment, which suppress or promote the growth of other plant species [1]. Allelochemicals from plants can interfere with the growth and development of conspecific individuals in a phenomenon called autotoxicity [2], where the allelochemicals released into the environment at high concentrations accumulate in the soil to affect the germination or growth/development of the plant itself or the next crop of the same species [3]. Autotoxicity has become a significant agricultural constraint, contributing to “replant problems” and “soil sickness” that limit the sustainability of continuous monocultures in various crops, including coffee (*Coffea arabica*) [4] and alfalfa (*Medicago sativa*) [2,5]. Autotoxicity has also been observed in certain ecosystems, including forests, grasslands, and orchards, where it causes several ecological and economic complications, such as a decline in crop yield, continuous cropping obstacles, regeneration failure of forests, and replant problem in orchards [2,6].

Among the economically vital tree crops, cashew (*Anacardium occidentale* L.) is of global importance. Although the plant is native to northeastern Brazil, it is now extensively cultivated across the tropics due to its socio-economic importance and high adaptability to diverse geographical regions [7,8,9]. Cashew is an evergreen perennial tree crop that grows rapidly, reaching an average height of about 5–8 m and even 20 m high and 12–14 m wide [9]. Due to the architecture of the plant, it was initially used as an agroforestry plant for land reclamation to boost productivity by avoiding desertification and soil erosion [10]. The tree is valued for its yield of nutritious, kidney-shaped nuts, a high-value global commodity rich in proteins and unsaturated fats, and the vitamin C-rich pseudofruit (cashew apple), used in juices, jams, and traditional medicine [8,11,12]. Globally, about 54% of the cashew nut production is attributed to Africa, with Côte d’Ivoire being the leading nut producer with an average amount of 7.3 million tons from 2018 to 2022. Next to Africa in terms of nut production is Asia, contributing 41.9%, and the Americas being the least with 4.1% [13]. Furthermore, the various parts of the cashew contain a diverse array of bioactive compounds, including anacardic acids, phenols, tannins, and flavonoids, which are linked to both its economic utility in industry and its documented use in traditional pharmacopeia [14].

In established cashew plantations, a notable reduction in understory vegetation is commonly observed [9,10]. Although a thick canopy in established plantations could be a factor, the reported allelopathic potential of cashew indicates there is a potential biochemical component. Extracts from cashew leaves, stem bark, and nutshell liquid have been shown to inhibit the germination and growth of various test species, including lettuce, cowpea, cucumber, and maize [12,15,16,17]. Despite its economic lifespan of 20–25 years and the known productivity declines in old cashew orchards, which are often attributed to pests and soil depletion [8,9,10], the potential role of autotoxicity in cashew replanting failure remains a critical knowledge gap. This is a significant oversight in research, as autotoxicity directly threatens the long-term viability and economic sustainability of cashew cultivation.

Therefore, to address this gap, the present study was designed to quantitatively evaluate the allelopathic potential of *A. occidentale* leaf and stem bark extracts on model plant species (lettuce, tomato, and pepper) under laboratory conditions, and to evaluate and provide the first explicit evidence for potential autotoxicity in cashew by evaluating the effects of cashew leaf amendments and aqueous extracts on the growth of cashew seedlings in a greenhouse. This research aims to determine whether autotoxicity could be a major biological barrier to cashew replanting, thereby informing future sustainable orchard management practices.

## 2. Results

### 2.1. Effect of A. occidentale Leaves and Stem Bark on the Radicle and Hypocotyl Elongations of Lettuce

**Figure 1:**
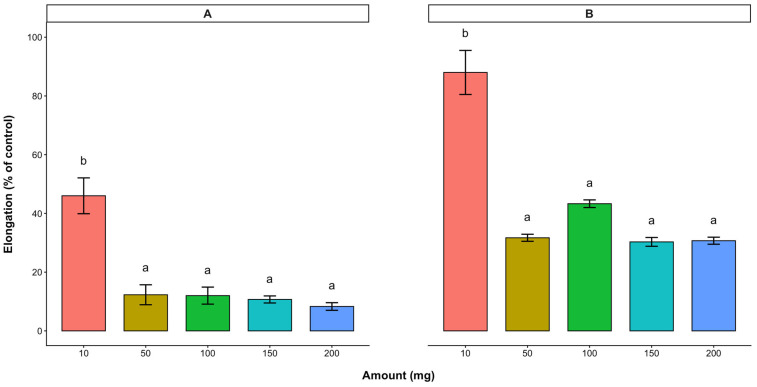
Effect of *A. occidentale* leaves on the radicle (**A**) and hypocotyl (**B**) elongations of lettuce. Values are means ± SD of three replications (*n* = 15). Significant variations between treatments are represented by different letters (according to Fisher’s LSD test at a 0.05 level of probability).

Oven-dried cashew leaves and stem bark were obtained from cashew plants to evaluate the effect of leachates on the radicle and hypocotyl elongations of lettuce using the Sandwich bioassay developed in [17]. Both cashew dried leaves and stem bark significantly inhibited the radicle and hypocotyl elongations with increasing amounts of the sample (Figure 1).

**Figure 2:**
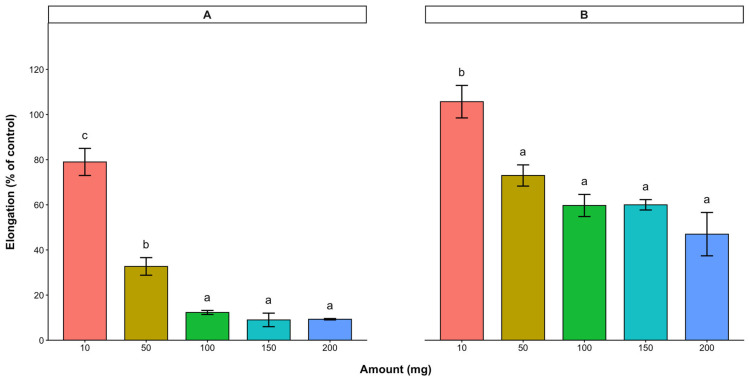
Effect of *A. occidentale* stem bark on the radicle (**A**) and hypocotyl (**B**) elongations of lettuce. Values are means ± SD of three replications (*n* = 15). Significant variations between treatments are represented by different letters (Fisher’s LSD test at a 0.05 level of probability).

With the leaf treatment, radicle and hypocotyl elongations were 46.0% and 88.3% (of the control), respectively, for the 10 mg dried cashew leaves. Although higher amounts (50–200 mg) significantly reduced radicle (8.3–12.2% of the control) and hypocotyl (30.3–43.3% of the control) elongation, no significant differences were observed among the treatments at these applied quantities. With the 10 mg cashew stem bark treatment, the radicle and hypocotyl elongations of lettuce were 79.2% and 105.7% of the control, respectively (Figure 2). Similar to the cashew leaves, higher amounts of the stem bark further reduced the lettuce radicle (9.3–32.7% of the control) and hypocotyl (45.3–73.1% of the control) elongation. In both the cashew leaves and stem bark, the radicle elongation of lettuce was inhibited more than the hypocotyl elongation.

### 2.2. Effect of A. occidentale Aqueous Leaf Extract and Root Exudates on the Germination of Lettuce, Tomato, and Pepper Seeds

Aqueous extract of cashew leaves was tested at varying concentration levels (0.5–50 mg/mL) on the germination of lettuce, pepper, and tomato seeds. The results showed a proportional increase in the inhibitory effect of cashew aqueous leaf extract on the germination of the test plants (Table 1). The germination of lettuce was completely inhibited at 50 mg/mL, whereas lower concentrations (0.5–10 mg/mL) did not exhibit any significant effect on germination. The germination of pepper was only affected (42%) at 50 mg/mL. On the contrary, cashew aqueous leaf extract did not have any effect on the germination of tomato seeds.

The root exudates collected from 7-week-old cashew seedlings were tested on the germination performance of lettuce, tomato, and pepper. The results of the study showed that cashew root exudate had no significant effect on the germination of the test plants (Table 2). Although the germination of pepper seeds was relatively low (56.7–71.3%) among the varying concentrations, there was no significant difference in the treatment effect.

### 2.3. Effects of A. occidentale Aqueous Leaf Extract on the Radicle and Hypocotyl Elongations of Lettuce, Tomato, and Pepper

**Figure 3:**
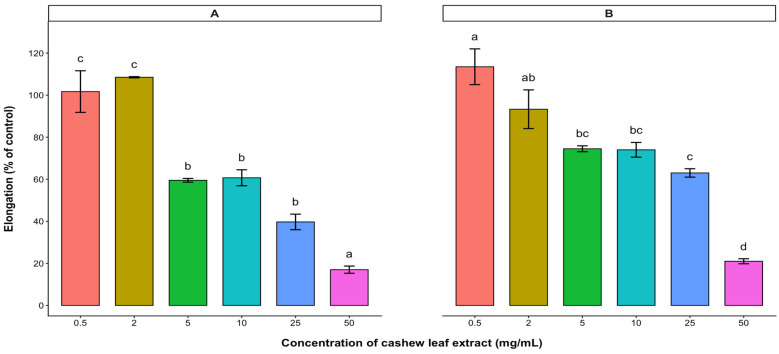
Effect of *A. occidentale* leaf extract on the radicle (**A**) and hypocotyl (**B**) elongations of lettuce. Values are means ± SD of three replications (*n* = 15). Significant variations between treatments are represented by different letters (according to Fisher’s LSD test at a 0.05 level of probability).

**Figure 4:**
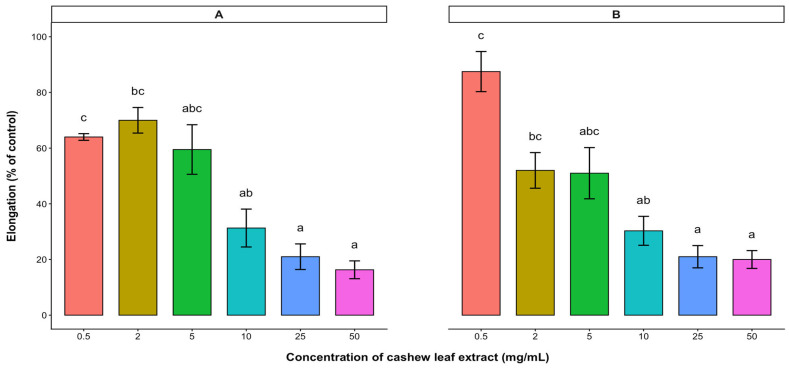
Effect of *A. occidentale* leaf extract on the radicle (**A**) and hypocotyl (**B**) elongations of tomato. Values are means ± SD of three replications (*n* = 15). Significant variations between treatments are represented by different letters (Fisher’s LSD test at a 0.05 level of probability).

**Figure 5:**
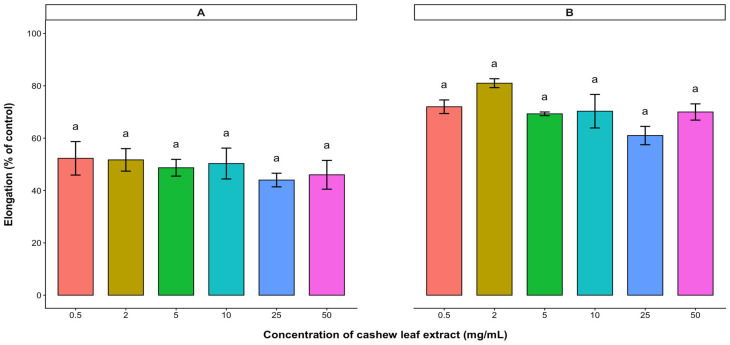
Effect of *A. occidentale* leaf extract on the radicle (**A**) and hypocotyl (**B**) elongations of pepper. Values are means ± SD of three replications (*n* = 15). Significant variations between treatments are represented by different letters (Fisher’s LSD test at a 0.05 level of probability).

The potential plant growth inhibitory effect of the water-soluble bioactive compounds contained in the aqueous leaf extract of cashew was evaluated on the radicle and hypocotyl elongations (expressed as % control) of the test crops. Based on the results, radicle and hypocotyl elongations of lettuce, tomato, and pepper were significantly reduced with increasing concentration of the aqueous leaf extract. In lettuce and tomato, radicle and hypocotyl elongations were inhibited in a dose-dependent manner, with radicle elongation being inhibited more than hypocotyl elongation (Figure 3 and Figure 4). At low concentrations (0.5–2 mg/mL), the extracts of cashew leaves did not affect the radicle elongation of lettuce (Figure 3). Both radicle and hypocotyl elongations of lettuce were reduced to less than 50% of the control at a 50 mg/mL treatment. However, the 10 mg/mL treatment reduced both radicle and hypocotyl elongations of tomato below 50% of the control (Figure 4). Although cashew aqueous leaf extract affected the radicle and hypocotyl elongations of pepper, there was no significant difference among the treatments (Figure 5).

### 2.4. Effects of Root Exudates of A. occidentale on the Radicle and Hypocotyl Elongations of Lettuce, Tomato, and Pepper

**Figure 6:**
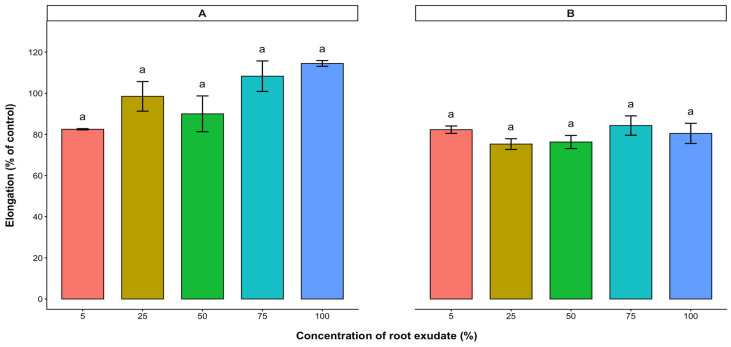
Effect of *A. occidentale* root exudate on the radicle (**A**) and hypocotyl (**B**) elongations of lettuce. Values are means ± SD of three replications (*n* = 15). Significant variations between treatments are represented by different letters (Fisher’s LSD test at a 0.05 level of probability).

**Figure 7:**
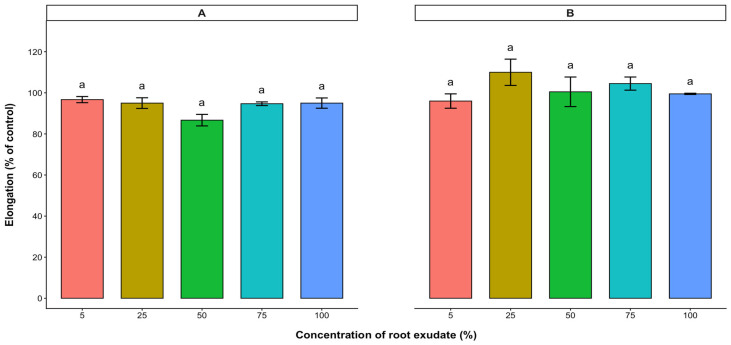
Effect of *A. occidentale* root exudate on the radicle (**A**) and hypocotyl (**B**) elongations of tomato. Values are means ± SD of three replications (*n* = 15). Significant variations between treatments are represented by different letters (Fisher’s LSD test at a 0.05 level of probability).

**Figure 8:**
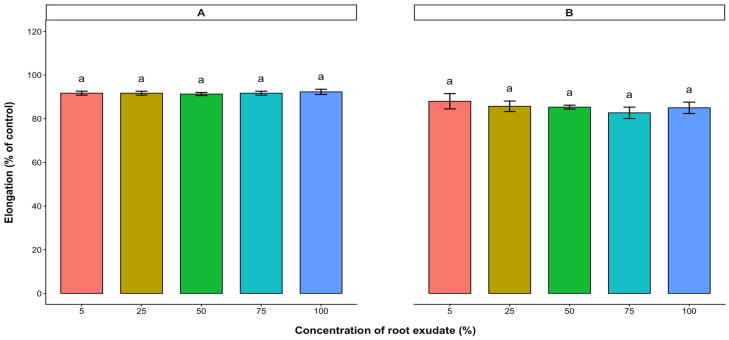
Effect of *A. occidentale* root exudate on the radicle (**A**) and hypocotyl (**B**) elongations of pepper. Values are means ± SD of three replications (*n* = 15). Significant variations between treatments are represented by different letters (Fisher’s LSD test at a 0.05 level of probability).

Root exudates collected from 7-week-old cashew seedlings were tested on the radicle and hypocotyl elongations of the test crop seeds. Varying concentration levels of root exudate tested showed no significant effect on the radicle and hypocotyl elongations of lettuce, tomato, and pepper (Figure 6, Figure 7 and Figure 8).

### 2.5. Evaluating the Potential Autotoxic Effect of Cashew Aqueous Leaf Extract and Powder on Cashew Seedlings

**Figure 9:**
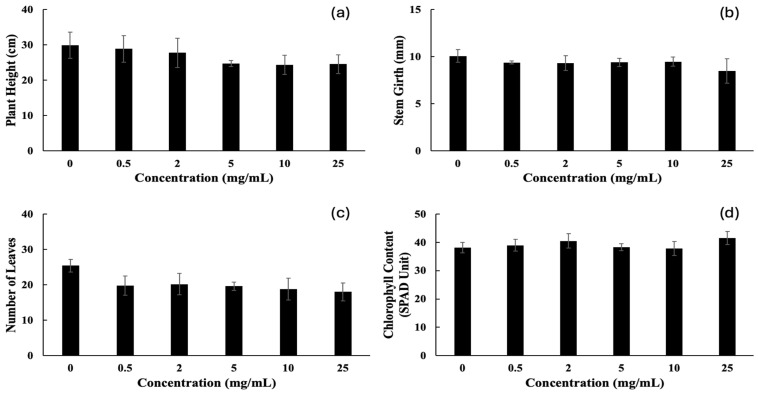
Plant height (**a**), stem girth (**b**), number of leaves (**c**), and chlorophyll content (**d**) of cashew seedlings treated with aqueous leaf extract of cashew for 13 weeks. Values are means ± SD of three replications (*n* = 15).

The potential autotoxic effect of water-soluble allelochemicals contained in the aqueous leaf extract of cashew was assessed on cashew seedlings in the greenhouse. The cashew aqueous leaf extract did not affect the plant height, stem girth, and chlorophyll content of cashew seedlings after 13 weeks of application (Figure 9). Although the aqueous leaf extract of cashew affected the number of leaves, there were no significant differences among the applied concentrations (Figure 9c). Consequently, the aqueous leaf extract of cashew did not have any effect on the biomass of cashew seedlings Table 3).

**Figure 10:**
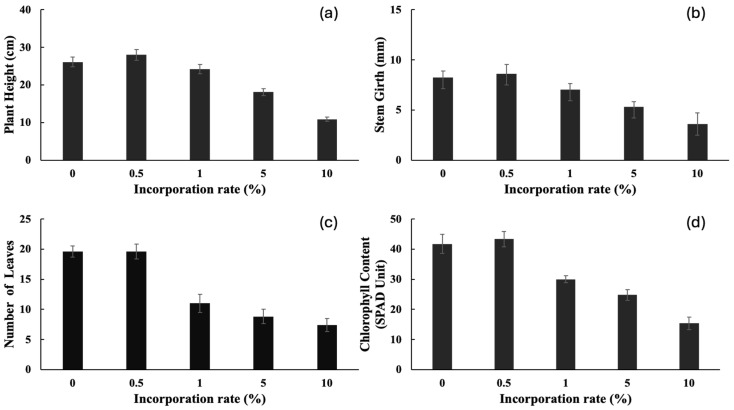
Plant height (**a**), stem girth (**b**), number of leaves (**c**), and chlorophyll content (**d**) of cashew seedlings treated with cashew leaf powder for 13 weeks. Values are means ± SD of three replications (*n* = 15).

**Figure 11:**
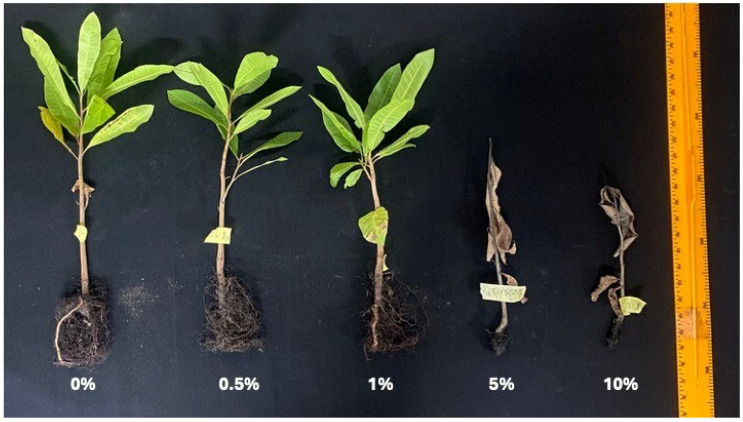
Cashew seedlings grown in soil incorporated with cashew leaf powder for 13 weeks.

Cashew leaf powder was incorporated into growing media to evaluate the autotoxic effect on cashew seedlings, mimicking the deposition of allelochemicals into the soil through litter fall in cashew plantations. The results showed significant inhibition of the growth parameters of the cashew seedlings (Figure 10). Senescence was observed in plants with high powder incorporation rate (5 and 10%), as shown in Figure 11. At a high incorporation rate (5–10%), the plant height (Figure 10a), stem girth (Figure 10b), number of leaves (Figure 10c), and chlorophyll content (Figure 10d) were significantly reduced compared to the control.

The results of plant biomass of cashew seedlings show significantly high inhibition with increasing rate of powder, hence resulting in low biomass values at 10%, including shoot length (4.50 cm), root length (2.20 cm), fresh shoot weight (3.70 g), fresh root weight (2.16 g), dry shoot weight (1.38 g), dry root weight (0.94 g), and root volume (1.00 mL), as shown in Table 4.

## 3. Discussion

### 3.1. Allelopathic Potential of A. occidentale Leaves, Stem Bark, and Leaf Extract

The results of this study demonstrate that leachates from cashew leaves and stem barks possess potential plant growth inhibitory activities. The radicle elongation of lettuce was more severely inhibited than hypocotyl elongation, a common response attributed to the direct exposure of root tissues to allelochemicals and their higher sensitivity during early developmental stages [18,19]. This aligns with findings by Fujii et al. [17], who reported similar inhibitory effects from oven-dried cashew leaves. The high susceptibility of radicle to inhibition suggests allelochemicals may primarily disrupt processes critical for root cell division, elongation, and higher porosity of the root surface, potentially impacting water and nutrient uptake in affected seedlings [18,19,20,21]. The vulnerability of lettuce radicle resulted in high inhibition, particularly at higher concentration levels tested in both cashew stem bark and leaves. This observation is similar to the findings in some allelopathic studies from [22,23].

Seed germination is an important phase in crop growth and development, which requires appropriate conditions for a successful establishment [24]. The species-specific response to aqueous leaf extracts, including the complete inhibition of lettuce germination, moderate inhibition of pepper, and no effect on tomato, highlights the selective nature of these allelochemicals. This variability in the inhibition of germination can be linked to differences in seed coat permeability and physiological tolerance among species [25]. As an example, the resilience of the tomato may be due to its suberin-rich seed coat acting as a barrier [26]. Ifediora and Nwokeocha [15] also reported that cashew aqueous leaf extract suppressed the germination of cowpea at the maximum concentration. Another pivotal finding of our work is the clear lack of inhibitory effect from the root exudates collected from the 7-week-old cashew seedlings on all the tested parameters across the three species. The lack of inhibitory effect from root exudates collected from young cashew seedlings suggests that, in early growth stages, allelochemical release occurs primarily through above-ground litter decomposition rather than rhizodeposition [27]. This is consistent with findings on other perennial species where phytotoxin accumulation is driven by litter fall [28]. Liu et al. [29] reported that the seed germination rate and seedling biomass of *Picea asperata* were stimulated, while those of *Betula albosinensis* and *Betula platyphylla* were suppressed by the root exudates of herbs (*Poa annua* and *Potentilla fragarioides*). Wang et al. [5] also reported findings on the inhibition of germination of *Medicago sativa* by the aqueous root extract with increasing concentration in *Medicago truncaluta*. The absence of activity in root exudates of cashew seedlings does not entirely rule out their role in mature trees, as the quality and quantity of exudates can change with plant age and environmental stress [30].

The effects of cashew aqueous leaf extract and root exudate were evaluated on the radicle and hypocotyl lengths of lettuce, tomato, and pepper. The results showed that cashew aqueous leaf extract significantly affected both the elongation of the hypocotyl and radicle of lettuce and pepper. Similar to the effects of the dried leaves and stem barks, the radicle elongation of lettuce was more highly inhibited than the hypocotyl elongation. Similarly, Ifediora and Nwokeocha [15] reported that increased concentrations of cashew aqueous extracts had distinct inhibitory effects on seed germination and germination rate of cowpea. Allelochemicals from allelopathic species negatively affected many cellular processes of the target plant by destroying the membrane porosity and ion intake [31,32,33], suppressing electron movement in their photosynthetic and respiratory pathway [34], destroying DNA and protein, changing some enzymatic activities [33,35], and eventually resulting in programmed cell senescence [36]. Although both the radicle and hypocotyl elongations of pepper were reduced below 80% of the control by cashew aqueous leaf extract, there was no notable variation among the various concentrations. This inhibition can be attributed to the existence of water-soluble compounds available in leaf extract [5].

### 3.2. A. occidentale Has Potential Autotoxic Effects

In an era where arable land for farming is difficult to acquire, it is up to agricultural scientists to intensify research on monoculture systems to avert the risk of continuous cropping systems, such as plants like *A. occidentale*. The most agriculturally significant result of this study is the report on the potential of autotoxicity in cashew. Continuous cropping in monoculture cropping systems can lead to the accumulation of allelochemicals that can affect the growth of the monocultured crop and re-establishment [5]. Cashew regularly releases some bioactive compounds that enter the soil via root exudate, leachates from leaves, and litter fall decomposition. These compounds released can directly or indirectly suppress seed germination, radicle elongation, and plant growth [37], causing low seed germination rates, poor seedling growth, and yield and quality reduction [38]. While the application of cashew aqueous leaf extracts did not affect cashew seedling growth, the incorporation of leaf powder (5–10% incorporation rate) into the growth medium caused severe dose-dependent inhibition across all measured parameters, including plant height, stem girth, leaf number, chlorophyll content, and biomass.

The plant height, stem girth, leaf number, and chlorophyll content of cashew seedlings were significantly reduced, leading to a reduction in overall biomass at the 5% and 10% incorporation rates of the dried leaves. The growth of cashew seedlings declined, and the biomass decreased notably at high concentrations (5% and 10%), showing a potential for autotoxicity. This indicates that autotoxicity is mediated through the gradual release of allelochemicals during leaf litter decomposition, creating a sustained phytotoxic environment in the rhizosphere [39].

The results of this study mirror autotoxicity mechanisms documented in other plant species, including alfalfa (*Medicago sativa*) [39], coffee (*Coffea arabica* L.) [4], and rice (*Oryza sativa* L.) [40], where accumulated leaf litter leads to “replant failure” and soil sickness [2,6]. The bioactive compounds identified in cashew leaves, including quercetin, epicatechin, chlorogenic acid, and gallic acid, are known allelochemicals [14,41]. For example, epicatechin has been linked to autotoxic effects in *Actinidia deliciosa* [42], while quercetin can disrupt photosynthesis and membrane integrity [43]. Gallic acid had the highest specific activity in the seeds of *Fagopyrum tataricum* [44], and is the main allelochemical found in the root exudates of reed, capable of inhibiting root growth in plants [45,46]. Chlorogenic acid is another compound found in the leaves of the cashew. Bengyella et al. [47] reported that chlorogenic acid affected the germination index (GI), seed vigor, fresh seedling weight, coleoptile length, primary root length, and root hairs of *Festuca arundinacea*. In the leaves of *Rosmarinus officinalis*, chlorogenic acid had a very low inhibitory effect on the radicle elongation of lettuce [48]. The gradual decomposition of cashew leaf litter could likely release a complex mixture of these compounds, which collectively inhibit the establishment and growth of conspecific seedlings.

### 3.3. Implications for Cashew Orchard Sustainability

The findings of this study on autotoxicity directly address a critical knowledge gap in cashew production. The decline in productivity of old orchards, often attributed to soil nutrient depletion or pests [9,10], could further be significantly exacerbated by autotoxicity. The demonstrated suppression of cashew seedling growth in litter-amended soil provides a plausible biological explanation for the challenges associated with replanting cashew in existing plantations. The reported autotoxic effect, mediated through litter accumulation, represents a key risk factor for the long-term sustainability of monoculture cashew production systems.

Moving forward, management strategies must consider litter management as a vital component of orchard regeneration. Practices such as litter removal, intercropping, or the use of activated charcoal to adsorb allelochemicals have proven effective in other autotoxic cropping systems [28,49] and should be investigated for cashew. Further research is also needed to identify the specific bioactive compounds responsible for autotoxicity.

## 4. Materials and Methods

### 4.1. Plant Materials

**Figure 12:**
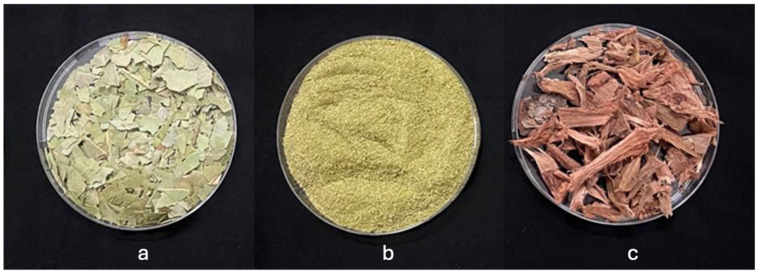
Dried cashew leaves (**a**), cashew leaves powder (**b**), and dried cashew stem bark (**c**) used in this study.

Cashew (*Anacardium occidentale* L.) plant samples, including nuts (seeds), leaves, and stem bark (Figure 12), were collected from the University of Ghana Research Farms (which lies approximately on latitude 5.6633° N and longitude 0.2035° W), and oven-dried at 60 °C for 72 h. The variety of cashew used in this study was the Brazilian variety, due to its dominance on the farms in Ghana. Receptor plants, including lettuce (*Lactuca sativa*) Eden variety, tomato (*Solanum lycopersicum*) Petomech+ variety purchased from an agro-input shop (Farmers Friend, Accra, Ghana), and pepper (*Capsicum annum*) Legon 18 variety (purchased from Crop Science Department, University of Ghana), and used for the bioassay, while raised cashew seedlings were used for the autotoxicity study. The seedlings were kept in a refrigerator until they were used for the bioassay.

### 4.2. Cashew Aqueous Leaf Extraction

The collected cashew leaves were washed to remove dirt and oven-dried at 60 °C for 72 h. The dried leaves were ground into powder and kept in a paper envelope. The powdered dried cashew leaves (25 g) were extracted with 500 mL of distilled water and shaken for 3 days at 250 oscillations. The cashew aqueous leaf extract was filtered through filter paper and diluted into varying concentrations (0.5–50 mg/mL) and used for the bioassay.

### 4.3. Root Exudate Collection from Cashew Seedlings

Cashew nuts were collected from the University of Ghana Research Farms and germinated in a seed tray. The germinated seeds were transplanted into a nursery bag filled with soil. After 3 months, the cashew seedlings were placed in a hydroponic container filled with 300 mL of distilled water for 21 days. Distilled water was regularly added to each container to maintain a constant volume of water. Each container had one seedling with six replicates. The aqueous solution in each container was collected and filtered through 3 layers of gauze. The volume of the root exudates accounted for 0, 5, 25, 50, 75, and 100% of the total volume, and was used for the bioassay.

### 4.4. Determination of Biological Activities of Dried Cashew Leaves/Stem Bark, Leaf Extract, and Root Exudate

#### 4.4.1. Growth Inhibitory Effect of Cashew Leaf and Stem Bark Leachates

Cashew leaves were harvested from the University of Ghana Research Farm. The leaves were washed and oven-dried at 60 °C for 72 h. The stem bark sample also followed the same procedure, except for washing. After drying, the leaves and stem bark were crushed and broken, respectively, into smaller pieces and kept in an envelope for later use. Then, 0.75% *w*/*v* agar media was prepared by dissolving 12 g agar powder in 1600 mL of distilled water and autoclaved for 30 min at 121 °C. A varying amount (10, 50, 100, 150, and 200 mg) of both cashew leaves and stem bark was weighed into a six-well multi-dish plastic plate. An initial 5 mL of agar media was added into each of the wells and later topped up with an extra 5 mL of agar media, hence sandwiching the leaves and stembark. The control did not contain any plant material except the agar. After media solidification, lettuce seeds, 5 each, were placed into each cell, sealed with parafilm, and incubated for 3 days at 25 °C. The control did not contain any plant material except the agar. After 3 days of incubation, radicle and hypocotyl lengths (mm) were measured and expressed as a percentage of the control.

#### 4.4.2. Effects of Cashew Aqueous Leaf Extract and Root Exudate on the Germination of Lettuce, Pepper, and Tomato

The stock solution of cashew leaf extract (50 mg/mL) was diluted and formulated into 0.5, 2, 5, 10, 25, and 50 mg/mL extracts, and distilled water was used as a control. Filter paper was placed in a Petri dish (9 cm in diameter), and 5 mL of distilled water or extracts of different concentrations was added, and three replicates were set for each treatment. Hundred seeds of each of the target crops (lettuce, pepper, and tomato) were placed into Petri dishes containing filter paper moist with different treatments. The setup was sealed with parafilm to prevent water loss and incubated at 25 °C with daily germination count. Germination was counted when the radicle broke through the seed coat and reached about 2 mm in length. Germination percentage was calculated using the formula below:

Germination (G) = (number of seeds germinated/total number of seeds plated) × 100

#### 4.4.3. Growth Inhibitory Bioassay of Cashew Leaf Extract and Root Exudate

The six-well multi-dish plastic plate was laid with filter paper (33 mm diameter). Then, 0.75 mL each of the different concentrations of cashew leaf extract (0.5, 2, 5, 10, 25, and 50 mg/mL) and root exudates (5, 25, 50, 75, and 100%) was pipetted into the cells in the multi-well plastic dish containing filter paper. Distilled water was used as the control for all the treatments. Five pre-germinated seeds (lettuce, pepper, and tomato) were placed into the cells and sealed with parafilm for a 3-day incubation period at 25 °C in a complete randomized design. Three replicates were set for each of the treatments. After 3 days of incubation, radicle and hypocotyl lengths (mm) were measured and expressed as a percentage of the control.

Elongation % = (radicle or hypocotyl length of treated/radicle or hypocotyl length of control) × 100

#### 4.4.4. Growth Inhibitory Effect of Cashew Leaf Powder and Extract

Soilless media (Jiffy substrate) were used as the growing media for this study. Cashew seeds collected from the University farm were soaked for 3 days. After 3 days, the soaked seeds were raised in seed trays for 2 months before treatment application. Since the seedling growth stage of a plant is sensitive to environmental changes [50], it is often used to study allelopathy and autotoxicity effects [51], and this study also selected the seedling stage to evaluate the potential of autotoxicity in cashew. Furthermore, 200 g of the soilless media was weighed into the nursery bags. Cashew leaf powder was added to the media at different incorporation rates (0.5, 1, 5, and 10%). Each treatment had six replicates, and the control treatment did not receive any cashew leaf powder. In a different setup, the cashew seedlings were sprayed with cashew leaf extract (0.5, 2, 5, 10, and 25 mg/mL). Each treatment was replicated 6 times, and the control was sprayed only with distilled water. The setup was observed for 13 weeks, with the aqueous extract treatment application carried out bi-weekly, and data collection was done every week. Data was collected on plant height, stem girth, chlorophyll content, and number of leaves. After the 13 weeks, biomass data of cashew seedlings were taken for both the cashew powder experiment and the aqueous extract experiment.

### 4.5. Statistical Analysis

The experimental data were statistically analyzed using GenStat statistical analysis software (12th edition) and R statistical software26.0 software.

## 5. Conclusions

In conclusion, the findings of this study demonstrate the plant growth inhibitory activity of *Anacardium occidentale* leaves and stem bark. Laboratory assays confirmed its strong allelopathic potential, with leaf and stem bark leachates significantly inhibiting lettuce seedling growth, which affected radicle elongation of receptor plants more than hypocotyl elongation. Furthermore, aqueous leaf extracts differentially affected seed germination, severely inhibiting lettuce and pepper with no effect on tomato seed germination. Most critically, this research provides the first evidence for potential autotoxicity in cashew, showing that the decomposition of its own leaf litter in soil severely inhibits the growth of cashew seedlings. This novel finding identifies autotoxicity as a likely biological factor underlying the replanting problem and yield decline in aging orchards. Future efforts must address this challenge through targeted research and innovative soil management to ensure the long-term sustainability of cashew cultivation.

## Figures and Tables

**Table 1:** Effect of cashew leaf extract on the germination of lettuce, pepper, and tomatoes.

Concentration (mg/mL)	Germination (%)		
	Lettuce	Tomato	Pepper
0	47.3 c	90.7	60.7 b
0.5	46.7 c	90.7	70.0 b
2	44.7 c	92.7	72.7 b
5	42.7 c	94.7	67.3 b
10	45.3 c	89.3	72.0 b
25	18.0 b	91.3	68.0 b
50	0.00 a	85.3	42.0 a
*p* value	<0.001	NS	0.002

Values are means ± SD of three replications (*n* = 15). Means per factor followed by the same letter in the column do not differ significantly (according to Fisher’s LSD test at a 0.05 level of probability). NS indicates Not Significant.

**Table 2:** Effect of cashew root exudate on the germination of lettuce, pepper, and tomatoes.

Concentration (%)	Germination (%)		
	Lettuce	Tomato	Pepper
0	94.0	93.3	66.0
5	95.3	96.7	71.3
25	92.7	91.3	56.7
50	93.3	96.7	66.0
75	89.3	91.3	60.0
100	93.3	94.0	60.0
*p* value	NS	NS	NS

Values are means of three replications (*n* = 100). Means per factor followed by the same letter in the column do not differ significantly (according to Fisher’s LSD test at a 0.05 level of probability). NS indicates Not Significant.

**Table 3:** Biomass of cashew seedlings treated with aqueous leaf extract of cashew for 13 weeks.

Concentration (mg/mL)	SL (cm)	RL (cm)	FSW (g)	FRW (g)	DSW (g)	DRW (g)	RV (mL)
0	28.8 a	13.7 a	25.5 a	16.02 a	9.68 a	5.50 a	12.4 a
0.5	29.0 a	15.1 a	23.3 a	14.00 a	9.10 a	4.82 a	15.8 a
2	28.6 a	13.8 a	22.1 a	10.84 a	8.40 a	4.28 a	10.4 a
5	23.5 a	12.9 a	16.8 a	9.58 a	6.16 a	3.42 a	9.20 a
10	23.7 a	15.7 a	21.6 a	12.88 a	8.30 a	4.46 a	11.0 a
25	28.6 a	18.0 a	21.5 a	11.74 a	8.50 a	4.00 a	10.0 a
*p* value	0.689	0.353	0.479	0.465	0.487	0.686	0.483

Shoot length (SL), root length (RL), fresh shoot weight (FSW), fresh root weight (FRW), dry shoot weight (DSW), dry root weight (DRW), and root volume (RV) of cashew seedlings applied with cashew leaf powder. Values are means of three replications (*n* = 15). Means with the same letter in a column are not significantly different from each other.

**Table 4:** Biomass of cashew seedlings grown in media incorporated with cashew powder.

Incorporation Rate (%)	SL (cm)	RL (cm)	FSW (g)	FRW (g)	DSW (g)	DRW (g)	RV (mL)
0	25.9 bc	12.4 bc	19.7 c	9.92 b	7.76 b	3.14 a	9.80 b
0.5	28.1 c	12.4 bc	17.1 bc	8.98 b	7.14 b	3.52 a	6.60 b
1	29.3 c	13.0 c	17.3 bc	10.88 b	7.12 b	4.40 a	10.40 b
5	15.9 b	7.60 b	9.60 ab	7.28 ab	3.82 ab	3.12 a	6.80 b
10	4.50 a	2.20 a	3.70 a	2.16 a	1.38 a	0.94 a	1.00 a
*p* > 0.05	<0.001	0.002	0.004	0.048	0.013	0.173	0.018

Shoot length (SL), root length (RL), fresh shoot weight (FSW), fresh root weight (FRW), dry shoot weight (DSW), dry root weight (DRW), and root volume (RV) of cashew seedlings applied with cashew leaf powder. Values are means of three replications (*n* = 15). Means with the same letter in a column are not significantly different from each other.

## Data Availability

The original data presented in this study are included in the article; further inquiries can be directed to the corresponding author.
